# Connecticut

**Published:** 1896-09

**Authors:** 


*Connecticut.* Danvers has had a lesson in the prevalence of tuberculosis among one of her prominent herds, and now joins in the general demand for protection from this danger.

From three to five hundred cattle are being examined weekly with tuberculin in the Nutmeg State.

Many of the largest milk-handling companies in this State are demanding of the producers certificates based upon the tuberculin-test as to the freedom of their herds from tuberculosis.

